# Dose and number of applications that maximize fungicide effective life exemplified by *Zymoseptoria tritici* on wheat – a model analysis

**DOI:** 10.1111/ppa.12558

**Published:** 2016-06-10

**Authors:** F. van den Berg, N. D. Paveley, F. van den Bosch

**Affiliations:** ^1^ Department of Computational and Systems BiologyRothamsted ResearchHarpendenAL5 2JQUK; ^2^ ADAS High MowthorpeDugglebyMaltonYO17 8BPUK

**Keywords:** effective life, fungicide resistance, healthy area duration, leaf blotch, selection ratio

## Abstract

Two key decisions that need to be taken about a fungicide treatment programme are (i) the number of applications that should be used per crop growing season, and (ii) the dosage that should be used in each application. There are two opposing considerations, with control efficacy improved by a higher number of applications and higher dose, and resistance management improved by a lower number of applications and lower dose. Resistance management aims to prolong the effective life of the fungicide, defined as the time between its introduction onto the market for use on the target pathogen, and the moment when effective control is lost due to a build‐up of fungicide resistance. Thus, the question is whether there are optimal combinations of dose rate and number of applications that both provide effective control and lead to a longer effective life. In this paper, it is shown how a range of spray programmes can be compared and optimal programmes selected. This is explored with *Zymoseptoria tritici* on wheat and a quinone outside inhibitor (QoI) fungicide. For this pathogen–fungicide combination, a single treatment provided effective control under the simulated disease pressure, but only if the application timing was optimal and the dose was close to the maximum permitted. Programmes with three applications were generally not optimal as they exerted too much selection for resistance. Two‐application fungicide programmes balanced effective control with reasonable flexibility of dose and application timing, and low resistance selection, leading to long effective lives of the fungicide.

## Introduction

When developing a fungicide application programme for a particular pathogen–crop–fungicide combination, there are two key considerations: (i) efficacy: the treatment programme needs to provide effective control of the pathogen, and (ii) resistance management: consideration should be given to the selection pressure exerted by an application programme on a pathogen to evolve resistance to the fungicide's mode of action (MOA). Both these considerations are of key importance to achieve durable control of the pathogen population. A general criterion to compare durability of fungicide application programmes is the effective life of the pathogen–fungicide combination. Effective life is defined as the time between the introduction of the fungicide onto the market for use on the target pathogen, and the moment when effective control is lost, under the treatment programme, due to the build‐up of fungicide resistance (van den Bosch & Gilligan, 2008; Hobbelen *et al*., 2011a,b).

Two key decisions to make for a fungicide treatment programme, are (i) the number of applications that should be used per crop growing season, and (ii) the dosage that should be used in the applications. From a resistance management perspective, a larger number of applications and/or a higher dose are expected to increase the rate of selection for fungicide resistance (van den Bosch *et al*., 2011, 2014a,b). From a disease control perspective, a larger number of applications and higher dosages are expected to improve control. Thus, the conditions required for effective resistance management are in opposition to those for effective disease control; however, there should be an optimal combination of the number of applications and dosage that leads to effective disease control with a long effective life.

Generic guidance by the Fungicide Resistance Action Committee (FRAC) recommends avoiding repetitive use of a single MOA, limiting the number of applications and optimizing the dose (Brent & Hollomon, 2007). Recent specific guidance limits the number of succinate dehydrogenase inhibitor (SDHI) fungicide applications on wheat to a maximum of two per growing season. Because there are opposing requirements for effective control and resistance management, guidance about the number of sprays and dose needs to be considered on a case‐by‐case basis.

The aim of this study was to use model simulations to find combinations of the number of applications and dosage that lead to effective disease control with a long effective life. The example used was the control of the wheat pathogen, *Zymoseptoria tritici*, by a quinone outside inhibitor (QoI) fungicide with a single MOA and where fungicide resistance is characterized by a single mutation (or other genetic change) in the pathogen conferring a high level of resistance. This was compared with the use of the QoI fungicide mixed with a low‐risk multisite‐acting fungicide.

## Materials and methods

### Model description

The model used has been described previously by van den Berg *et al*. (2013), where a detailed description of the model equations and the parameter estimation can be found. The model extensions required to study the effects of fungicide mixtures are described in Data S1. Here, the crop and pathogen biology incorporated in the model are described, sufficient for the reader to follow the model analysis and its interpretation.

The model simulates epidemics caused by *Z. tritici* on the upper canopy of winter wheat. Leaf area occupied by resistant and sensitive strains is tracked to quantify changes in the resistant fraction (selection). The effects of fungicide treatments on the epidemic are simulated via their effects on pathogen life cycle components (infection, latent period and sporulation). The model simulates several successive seasons until effective control is lost.

#### Time

The timescale is temperature sum accumulated after 1 January, to account for the temperature dependence of both crop and pathogen dynamics (Trudgil *et al*., 2005). The seasonal temperature variations at Cambridge, UK (which is located in a major wheat‐growing region) during 1984–2003 are used, with an average temperature of 15.2 °C (Met Office, UK, published online).

#### Canopy dynamics

**Figure 1:**
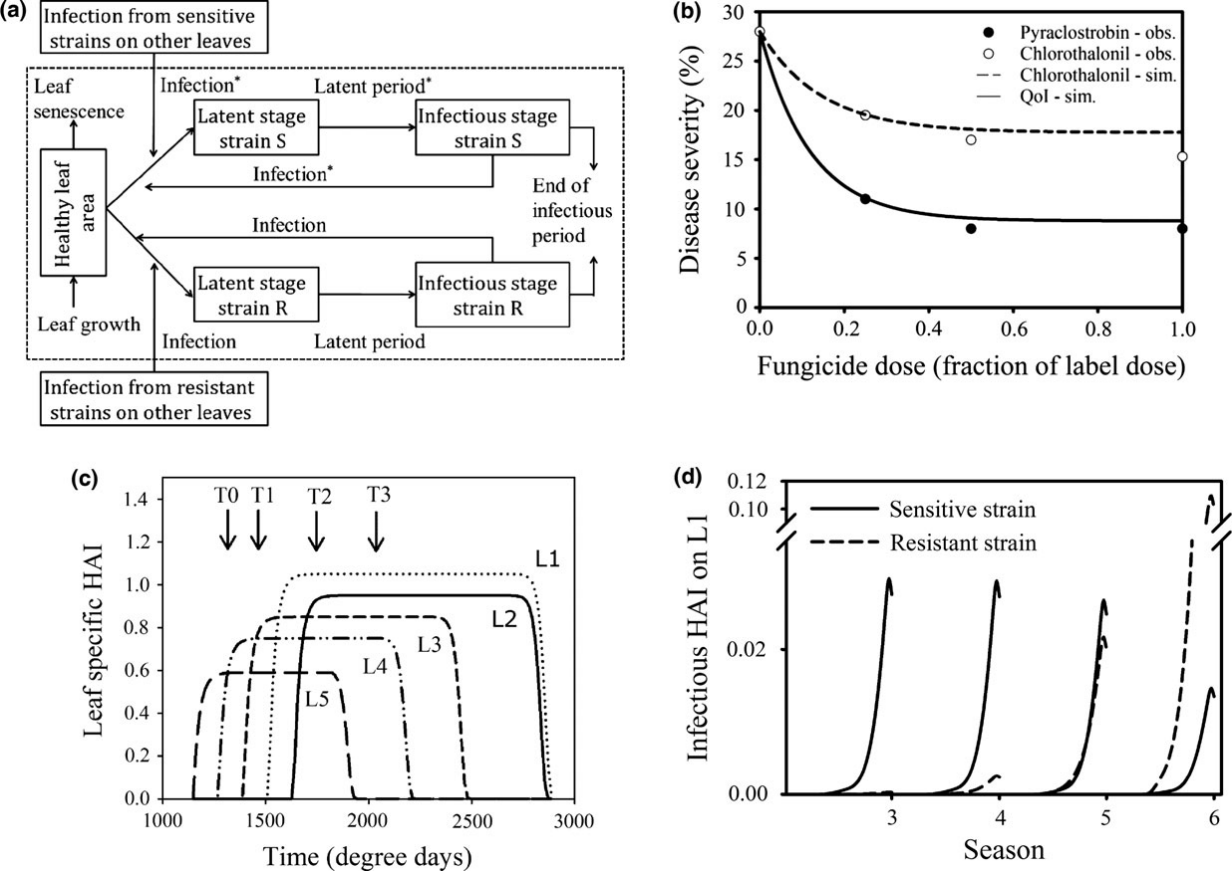
(a) Structure of the simulation model describing the development of fungicide‐sensitive and ‐resistant strains of *Zymoseptoria tritici* on a winter wheat leaf layer of the crop canopy during the host growing season. Strain S represents the sensitive pathogen strain and strain R represents the resistant pathogen strain. Life cycle traits that are affected by fungicide applications are marked with an *. (b) Reduction in disease severity at different doses of chlorothalonil, and pyraclostrobin used against *Zymoseptoria tritici*. The symbols represent field data from Lockley & Clark (2005), whereas the lines represent the simulated dose–response curves. (c) Graphical representation of the healthy area index (HAI) dynamics for the top five leaf layers of the wheat canopy, when uninfected, and the spray timings. Leaves are counted from the top down such that L1 represents the flag leaf. T0, T1, T2 and T3 refer to single fungicide spray applications at the full emergence of leaf four, leaf three, the flag leaf and at ear emergence, respectively. (d) Infectious area dynamics for the sensitive and resistant strain when sprayed each year with a full label dose at both T1 and T2.

The model tracks the growth of each of the individual top five leaves of a wheat crop canopy. Culm leaves are counted down the shoot, such that the flag (uppermost) leaf is referred to as leaf 1. A simulation season starts when leaf 5 becomes visible, i.e. at 1147 degree‐days. Leaves are assumed to grow at a constant rate until they reach their maximum size (Fig. 1c). The lag period between full leaf emergence and the onset of senescence is said to vary between 4 and 9 phyllochron (*P*) and to depend on leaf size (Lawless *et al*., 2005), whereby the largest leaf (leaf 2) is assumed to have a lag period of 9*P* (1098 degree‐days). At the end of the leaf's lifespan, the leaves start to senesce leading to a decrease in the healthy area index (HAI; m^2^ leaf area per m^2^ ground area) of the upper canopy (Fig. 1c). When a new leaf emerges it remains at the same height as the previously emerged leaf until it has fully emerged, after which it grows upwards over the internode distance (Lovell *et al*., 2004; Audsley *et al*., 2005), decreasing the vertical spore exchange between leaf layers.

#### Pathogen life cycle dynamics

In the model, the pathogen population consists of a strain that is sensitive to the fungicide applied and a strain that is resistant to the fungicide. Infection by a pathogen initially results in latent infection of leaf tissue. At the end of the latent period, there is sporulation throughout the infectious period after which it no longer contributes to the epidemic development (Fig. 1a). Because *Z. tritici* is a hemibiotroph, leaf senescence does not result in infectious lesion death but does remove the latent infections. Lesions on the rosette leaves, at the base of the canopy, are the main source of inoculum for the upper leaves 5 to 1 (Shaw & Royle, 1993).

The pathogen spores are distributed between the leaf layers by rain splash both upward and downward, and the probability that spores reach a particular leaf layer decreases exponentially with the distance from the inoculum source (Shaw, 1987). The temporary close proximity between leaf layers for a short period during and after leaf emergence results in increased spore transfer (Lovell *et al*., 2004). The overall transmission success of the pathogen spores incorporates the fact that the majority of spores do not land back on a plant, the probability that spores that do land back on a plant land on a healthy site, and the infection efficiency. The sensitive and resistant pathogen strains compete for space on healthy host tissues, with both density‐dependent and density‐independent periods of selection occurring within a host growing season. In epidemiological terms, density‐independent selection refers to the case where strain densities do not affect the pathogen transmission rates, which implies that new infections do not compete for space (van den Bosch & Gilligan, 2008); this tends to occur early on in the growing season when the overall densities are low. Later in the season, when the densities increase, the strains start to compete for space, leading to density‐dependent selection.

#### Fungicide dynamics

In the model, fungicide treatments affect the density of the pathogen strains through the pathogen's life cycle components. Two types of fungicide are considered: the first is a high resistance risk, single‐site acting systemic fungicide (hereafter referred to as ‘high‐risk’), affecting both the transmission rate and the length of the latent period of the sensitive strain. The second fungicide considered is a low resistance risk, multisite acting protectant fungicide (hereafter referred to as ‘low‐risk’), affecting the infection efficiency of both the sensitive and the resistant strain, but not affecting the latent period (Data S1). In the simulations, resistance evolves in response to the high‐risk fungicide, but there is no resistance against the low‐risk fungicide during the period simulated. It is assumed that the low‐risk and high‐risk fungicides have independent modes of action leading to a multiplicative survival effect of the two fungicides on the transmission rate of the sensitive strain (Hobbelen *et al*., 2011b) when both fungicides are present in a leaf. Only lesions that are in the first half of their latent period are affected by the eradicant action of the high‐risk fungicide (Paveley *et al*., 2012). The amount of fungicide that arrives at a given leaf depends on the leaf position within the crop canopy and the extent to which the leaf has emerged at the time of application (Milne *et al*., 2007). Therefore, part of the pathogen population, especially lower down in the host canopy, might largely escape fungicide treatment. From the time of application, the fungicide will start to decay leading to a reduction in efficacy of the applied active substance of the fungicide. The total amount of fungicide landing on a leaf, leaf size and the fungicide decay rate subsequently determine the effective dose concentration on the leaf, and hence the effect on the life cycle component(s).

#### Primary inoculum

The initial source of inoculum initiating the simulated epidemic consists of ascospores landing on the rosette leaves. A fraction of the lesions on the rosette leaves resulting from infection by these ascospores is resistant. It is assumed that the fraction of resistant infectious lesion tissues, at the end of year *n* is equal to the fraction of resistant lesions on the rosette leaves in year *n *+* *1. The fraction of resistance at the beginning of the first growing season is predefined and the effect of varying this initial fraction is explored.

### The specific case of *Z. tritici* on wheat

Although the model used in this paper is generic and can be applied to a range of cereal–pathogen systems, it is used here to consider the case of *Z. tritici* infecting wheat treated with (i) a systemic high‐risk single‐site fungicide to which the pathogen develops resistance through a single mutation and which has both protectant and eradicant action affecting only the sensitive strain; and (ii) a mixture of the high‐risk fungicide with a nonsystemic low‐risk multisite fungicide to which the pathogen does not develop resistance over the timescale under consideration and which only has protectant action that affects both the sensitive and resistant strain. The model was parameterized using field data from studies using the high‐risk QoI fungicide pyraclostrobin and the low‐risk fungicide chlorothalonil (Lockley & Clark, 2005; Fig. 1b; see Data S2 for further explanation).

Current practice for the control of *Z. tritici* in the UK is to apply two sprays of up to the label dose; one at the full emergence of the third leaf down the canopy (termed a ‘T1’ spray) followed by a second spray at the full emergence of the flag leaf (‘T2’ spray). On cultivars with good host resistance, this usually provides adequate disease control under low or average disease pressure. However, in the case of more severe outbreaks, additional applications might be required at the emergence of leaf 4 (‘T0’ spray) and/or ear emergence (‘T3’ spray) (Fig. 1; Oxley & Burnett, 2008; Clark, 2011; Paveley *et al*., 2012).

### Output variables

Three main outputs are:

1. Healthy area duration (HAD). Waggoner & Berger (1987) calculated this as the total area under the healthy and latent leaf area index curves for leaves 1–3 integrated over the grain filling period, i.e. between 2150 and 2900 degree‐days. Here, the latently infected tissues are included in HAD because, for the hemibiotrophic pathogen *Z. tritici*, these tissues still contribute to photosynthesis.
2. Selection ratio, defined as the proportional increase in the frequency of the resistant strain on the top five leaves during one full growing season.
3. Fungicide effective life, defined as the number of consecutive growing seasons that the fungicide (as solo product or in mixture) is able to maintain ‘effective control’, which is defined as keeping the disease‐induced HAD loss below a predefined threshold of 5% (Hobbelen *et al*., 2011b).

Both the HAD losses and selection ratios are presented for the first growing season, during which HAD is largely unaffected by the presence of the resistant strain and the selection ratio is largely unaffected by competition between resistant lesions. Hence, they provide a good quantification of efficacy and selection, and are indicative for the eventual effective lives.

### Simulations

The main questions being addressed are whether there is an optimal combination of dose and number of applications that maximizes effective life, and whether the optimal strategy is affected by factors such as the initial fraction of resistance, spray timings, the temporal proximity of spray applications or the presence of a low‐risk fungicide as a mixing partner. All possible combinations of one‐, two‐, three‐ and four‐spray programmes at the T0, T1, T2 and T3 fixed spray timings are considered. The dose of each spray application is varied, but individual applications never exceed the maximum permitted label dose and the total dose applied during one growing season never exceeds twice the label dose per application for each fungicide (which is often defined as the maximum total dose on the product label) unless otherwise specified. Figure 1c provides a graphical representation of the spray timings in relation to leaf growth and senescence.

## Results

### Spray applications containing a single high‐risk fungicide

#### The selection ratio

**Figure 2:**
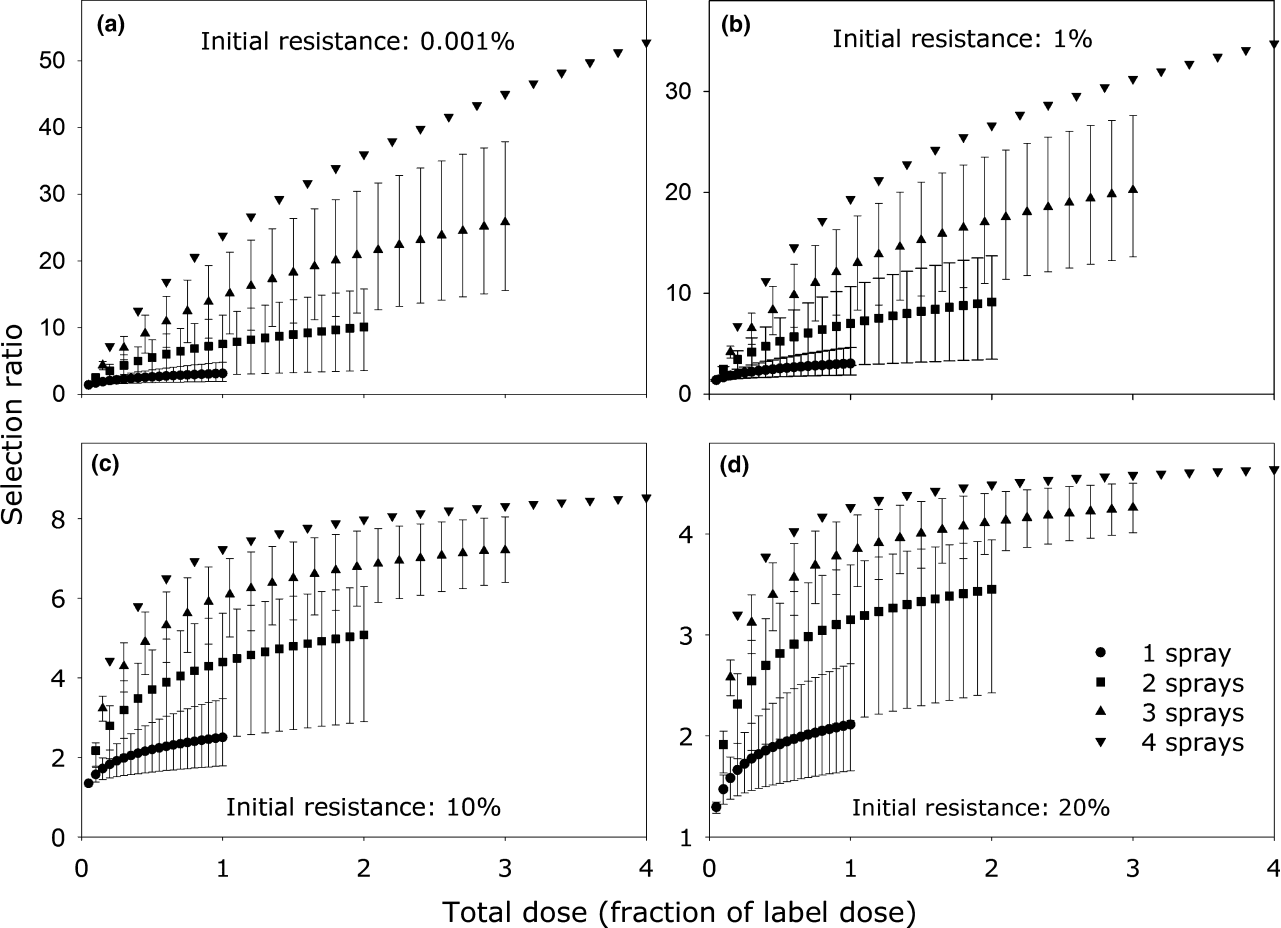
The effect on the selection ratio of the total high‐risk fungicide dose and the number of treatments over which the total dose is applied, for different initial fractions of resistance: (a) 0.00001 (0.001%); (b) 0.01 (1%); (c) 0.1 (10%) and (d) 0.2 (20%). The total dose listed on the *x*‐axis divided by the number of applications defines the dose applied per treatment. The dose of an individual application never exceeded the label dose. Note however, that in the case of the three‐ and four‐spray programmes and for high individual application doses the total dose applied during one growing season exceeds the maximum total dose on the product label (twice the label dose). Points represent the mean selection ratio for all possible combinations of the number of sprays within the group and the upper and lower bars represent the spray timing combinations (combinations of T0, T1, T2 and T3) leading to the minimum and maximum selection ratios for each group, respectively.

Figure 2 shows that, for a fixed number of applications, the selection ratio increases with the total dose in the spray programme. The relationship between the selection ratio and the total dose applied has a lower asymptote for large initial fractions of resistance. This is because the maximum possible selection ratio is the inverse of the initial fraction of resistance.

Holding the total dose of the spray programme constant, the selection ratio increases with the number of spray applications. However, there is some overlap between the maximum and minimum selection ratios of the different number of spray treatments, so, for example, the highest selection ratios (Fig. 2, upper bars) of the two‐spray programme are higher than the lowest selection ratios (Fig. 2, lower bars) associated with the three‐spray programme.

**Figure 3:**
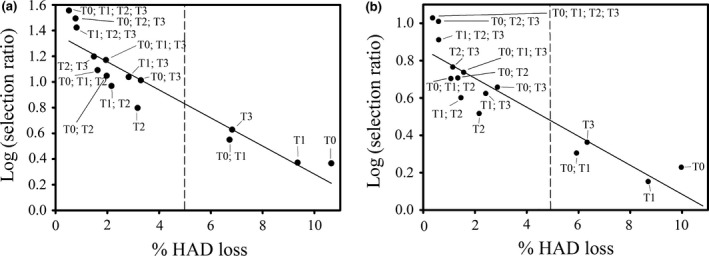
Selection ratio (log scale) versus percentage healthy area duration (HAD) loss experienced in the first host growing season for all possible spray timing combinations. (a) A solo high‐risk fungicide (QoI) is applied at each spray timing. (b) A 1:1 mixture of a high risk (QoI) and a low‐risk fungicide (chlorothalonil) is applied at each spray timing. The dose at individual spray timings within the chosen spray programme is adjusted such that the total dose applied during the complete growing season is always twice the label dose. Note that this means that for the single‐spray programmes the maximum dose allowed per application is exceeded. The solid lines were obtained by linear regression through all data points. Spray programmes that fall below these lines have a lower selection rate for fungicide resistance, at a given HAD loss, than other spray programmes and are therefore likely to result in a longer effective life. The dashed line represents 5% HAD loss, the effective control threshold.

Plotting the % HAD loss due to pathogen infection in the first year of the simulation (low HAD loss indicating high fungicide efficacy) against the selection ratio (log scale) in the first year, all spray programmes are scattered around a straight line (Fig. 3a). The figure clearly shows that there is a close correlation between disease damage, measured in HAD loss, and the selection pressure imposed by the fungicide to select for resistance. The figure also shows that some of the spray programmes clearly fall below this line. These spray programmes, such as the single T2 spray, the T1 + T2 spray programme and the T0 + T2 spray programme, have a lower selection rate for fungicide resistance at a given HAD loss, than other spray programmes. These programmes are therefore candidates for a longer effective life than the spray programmes that fall on the straight line or are above this line.

#### Fungicide effective life

**Figure 4:**
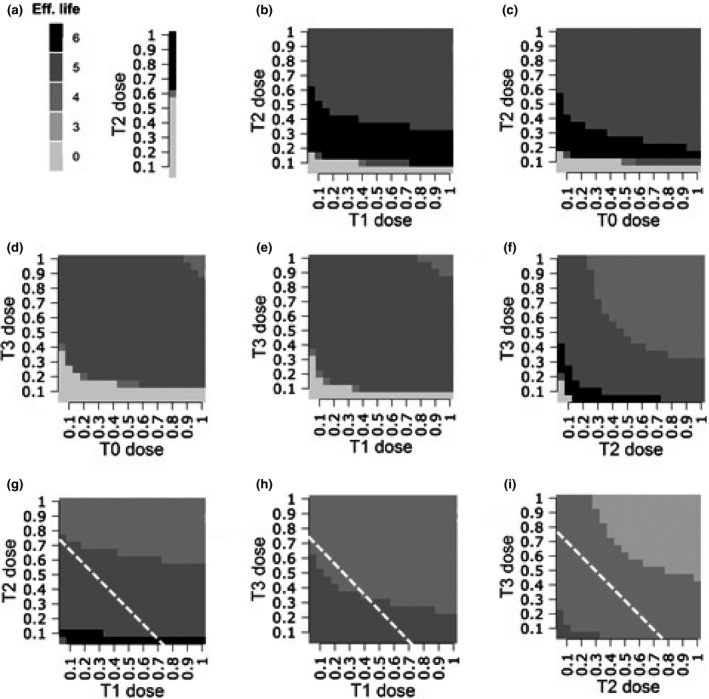
Effect of spray application dose of a high‐risk fungicide on fungicide effective life for application programmes of (a) a single T2 spray; (b) T1 + T2 sprays; (c) T0 + T2 sprays; (d) T0 + T3 sprays; (e) T1 + T3 sprays; (f) T2 + T3 sprays; (g) T0 + T1 + T2 sprays; (h) T0 + T1 + T3 sprays; and (i) T0 + T2 + T3 sprays. In the three‐spray programmes (g,h,i) the T0 dose is kept constant at 0.5 times the label dose. An effective life of zero refers to the situation when disease control is not adequate in the first growing season and the HAD loss exceeds the predefined loss threshold of 5%. All fungicide doses are given as a fraction of the label dose. The maximum dose allowed per spray application is one label dose and the total dose allowed per season is twice the label dose. Dose combinations that exceed this threshold lie above the dashed line.

Figure 4 corroborates the interpretation of the findings from Figure 3a. The spray programmes single T2, T0 + T2 and T1 + T2 give the longest effective life over a wide dose range of the sprays. The figure clearly shows that three‐spray programmes have a shorter effective life than the two‐spray programmes. All one‐spray programmes, except for the single T2 spray programme, have an effective life of zero years because the programme is not able to provide effective control even without resistance being present in the pathogen population.

Figure 4 shows that a T2 spray in an application programme is crucially important to achieve the longest effective fungicide life. Including a T3 spray lowers the effective life. The T1 + T2 spray programme has the largest dose range for which the maximum effective life is realized.

### Sprays containing a mixture of a high‐risk and a low‐risk fungicide

For fungicide mixtures where the low‐risk:high‐risk dose ratio is kept constant, both within and between seasons, the relation between % HAD loss and the selection ratio (log scale, Fig. 3b) is qualitatively similar to that for the solo product (Fig. 3a). The selection ratios are smaller, which reflects the effect that fungicide mixtures have on selection for resistance (van den Bosch *et al*., 2014a,b). For the 1:1 mixtures, the single T2 spray and the T1 + T2 spray programmes have the lowest selection ratio for the % HAD loss. This is very comparable to the situation with the solo fungicide, except that the T0 + T2 spray is much closer to the mean trend (the drawn line) with the mixture than where only a solo QoI spray is used. Because the spray programmes single T2 and T1 + T2 result in low selection for fungicide‐resistant strains, these programmes seem the best to maximize the effective life of the fungicide.

**Figure 5:**
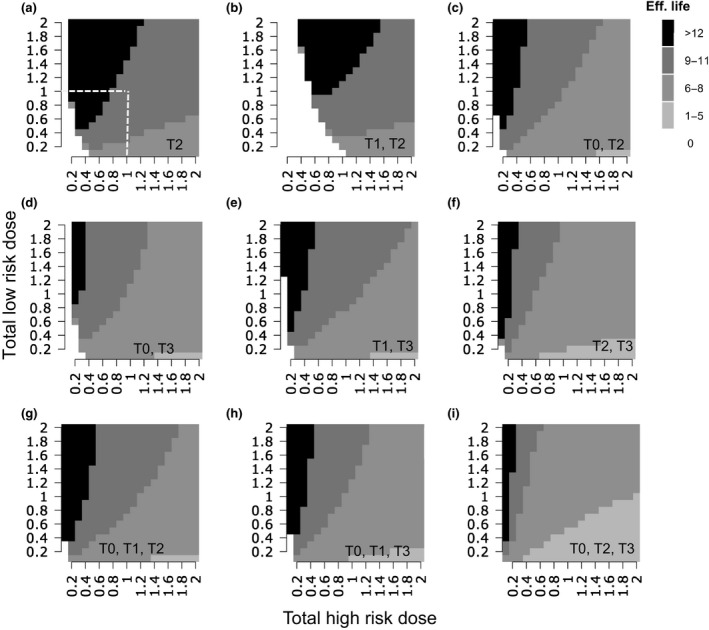
Effect of spray application dose of the high‐risk fungicide and low‐risk fungicide on fungicide effective life for application programmes of (a) a single T2 spray; (b) T1 + T2 sprays; (c) T0 + T2 sprays; (d) T0 + T3 sprays; (e) T1 + T3 sprays; (f) T2 + T3 sprays; (g) T0 + T1 + T2 sprays; (h) T0 + T1 + T3 sprays; and (i) T0 + T2 + T3 sprays. The total dose marked on the *x*‐axis is equally divided over the total number of spray applications present in the spray programme of interest. An effective life of zero refers to the situation when disease control is not adequate in the first growing season and the HAD loss exceeds the predefined loss threshold of 5%. All fungicide doses are given as a fraction of the label dose. The maximum dose allowed per spray application is one label dose and the total dose allowed per season is twice the label dose. Dose combinations that exceed this threshold lie above the dashed line.

Figure 5 explores, under constant total dose for the spray programme, how different spray programmes and mixtures of the low‐ and high‐risk fungicides influence the effective life. For any spray programme, the larger the dose of the low‐risk fungicide the larger the effective life of the fungicide. The high‐risk fungicide has clear dose limitations for the maximum effective life to be reached. This is in line with previous findings (van den Bosch *et al*., 2014b).

The single T2 and the T1 + T2 spray programmes show a wide dose range over which the maximum effective life is achieved. All other spray programmes have a narrower dose range for maximum effective life. For both mixture and solo fungicide spray programmes, a T2 spray is crucial for long effective life, and three‐ or four‐spray programmes are inferior to the two‐spray and the single T2 spray programmes.

## Discussion

For a range of pathosystems, the loss rate of fungicide efficacy due to resistance threatens to exceed the rate of introduction of new MOAs to the market (Russell, 2005; Cools & Fraaije, 2008; Stammler *et al*., 2008). It is therefore essential to develop effective fungicide resistance management methods. Approaches that have previously been proposed to slow down the rate of fungicide resistance build‐up are: a reduced application dose, a constraint on the number of applications, mixtures of MOAs, alternation of MOAs and adjustment of the application timing (Brent & Hollomon, 2007). However, these resistance management methods tend to be studied in isolation. Therefore, this study evaluates how the choice of application dose and the number of spray applications are best combined to optimize fungicide resistance management. The results were analysed for sprays containing only a high‐risk, single‐site acting fungicide and sprays in which the high‐risk fungicide is combined with a low‐risk mixing partner.

Both field (Fraaije *et al*., 2006; Mavroeidi & Shaw, 2006) and model studies (van den Bosch *et al*., 2011) have shown that increases in total fungicide dose increase the build‐up of resistance. There are also several experimental studies that have shown that splitting a fixed total fungicide dose over an increased number of spray applications leads to an increase in selection for fungicide resistance (Forester *et al*., 1994; Schulz, 1994; Engels *et al*., 1996; Metcalfe *et al*., 2000). These findings were consistent across a range of pathogen and host species, with disease control achieved by fungicides with different MOAs. The present model results are in accordance with these studies and reveal a clear relationship of increased selection ratios with both an increased total fungicide dose and an increased number of spray treatments.

The findings also agree with the governing principles for fungicide resistance management, as developed by van den Bosch *et al*. (2014a,b). Those authors state that the aim of fungicide resistance management is to reduce the product of the selection coefficient and fungicide exposure time. Both a reduction in dose and a reduction in the number of spray applications do just that. For example, a decreased dose reduces the per capita rate of increase of the resistant strain relative to that of the sensitive strain and thus reduces selection for fungicide resistance (Milgroom *et al*., 1989; van den Bosch *et al*., 2014a). On the other hand, a reduction in the number of spray applications reduces the exposure time, which also reduces the overall selection for fungicide resistance (Staub & Sozzi, 1983; van den Bosch *et al*., 2014a).

The question remaining is which combination of the two resistance management approaches is optimal. The governing principles laid out by van den Bosch *et al*. (2014a,b) suggest that combinations of dose and number of spray applications that have the same product of selection coefficient and exposure time will be equal with regards to resistance management. Therefore, the expectation is that there will be several optimal combinations of total fungicide dose and number of spray applications that maximize fungicide effective life, as found with the model analysis in the present study and is discussed below.

When sprays contain only a high‐risk, single‐site acting fungicide there is no single optimal combination of total fungicide dose and number of spray applications that maximizes fungicide effective life. Instead, the maximum effective life can be achieved by several different strategies: a single T2 spray, a T0 + T2 spray programme, a T1 + T2 spray programme or a T2 + T3 spray programme. Note that all these programmes contain a T2 spray, which is a particularly effective timing for preventing HAD loss from the upper canopy during the grain‐filling period (Paveley *et al*., 2012). However, some of these programmes require that the dose of fungicide be selected from a very narrow range to obtain the desired combination of efficacy and durability. With all the variability and uncertainty in disease severity and responses to fungicide applications in the field (te Beest *et al*., 2013) it would be best to select the spray programme from those that allow a wide dose range to achieve the maximum effective life. This reduces the choice of spray programme to a single T2 or a T1 + T2 spray programme. Applying more than two sprays per growing season of the high‐risk fungicide will not achieve the maximum effective fungicide life.

The single T2 spray programme, with the fungicide applied at full emergence of the flag leaf, has a relatively large dose range within which the maximum effective life can be achieved. However, this requires a higher total fungicide dose. Furthermore, this single spray programme has been shown to be sensitive to spray timings (van den Berg *et al*., 2013). If the spray timings need to be adjusted, for example, when the farmer has to delay the fungicide application due to unfavourable weather conditions, there is a high risk that these altered timings result in insufficient disease control (Paveley *et al*., 2000). Therefore, the T1 + T2 two‐spray programme is more robust than the other programmes that achieve maximum fungicide effective life.

Forester *et al*. (1994) stated that, in the case of split applications, the margin of error for the timings of individual applications is much smaller, but that this simultaneously leads to a much‐increased risk for the build‐up of resistance. Based on this, they suggest that, in agreement with the findings of the present investigation, a two‐spray programme rather than a single‐spray programme or a multispray programme is likely to be optimal to delay the build‐up of fenpropimorph resistance in powdery mildew.

Much of the discussion for solo fungicide treatment programmes also applies to the mixed treatment programmes, where a low‐risk fungicide is combined with the high‐risk fungicide. As found in a large number of studies, selection for fungicide resistance is strongly correlated with effectiveness of disease control (Hobbelen *et al*., 2011b, 2013; van den Bosch *et al*., 2014a,b). The two‐spray mixture programmes, as well as the single T2 mixture programme, achieve the maximum effective life over a larger dose range than the three‐spray programmes, and the largest dose ranges for maximum effective life are found for the single T2 and the T1 + T2 spray programmes. Thus, the results for mixture spray programmes lead to the same optimal spray programmes as with the solo fungicide, but longer effective lives are obtained by the use of mixtures.

The governing principles developed by van den Bosch *et al*. (2014a) can again be used to gain further insights into the results found. The low‐risk mixing fungicide has the same effect on the fitness of both the resistant and the sensitive strain; this results in a reduction of the selection coefficient and hence a reduction in selection for fungicide resistance. This overall reduction in selection pressure explains why the MOA mixtures lead to higher effective lives than sprays containing a single high‐risk MOA, even with similar combinations of dose and number of applications. Moreover, because the low‐risk mixing fungicide affects both strains equally, it is to be expected that the same application programmes for the single product sprays and the MOA mixture result in a similar pattern of change in the epidemic intrinsic growth rate of the sensitive strain. Thus, similar optimum combinations of dose and number of applications are required to maximize effective life.

The model simulations in the present study were run for the specific case of *Z. tritici* on wheat with QoI fungicides, alone or mixed with a low‐risk fungicide. However, the results are more generally applicable. As discussed in van den Bosch *et al*. (2014a,b), resistance evolution is driven by the difference in fitness between the fungicide sensitive and resistant strain, whereby fitness is expressed in terms of the epidemic intrinsic growth rate. Hence, in principle, pathosystems sharing similar patterns of change in the epidemic intrinsic growth rate with dose and number of applications are likely to share similar patterns of response to application programmes.

This study has shown how to evaluate a wide range of possible fungicide application programmes for their combined effectiveness in disease control and their selection for fungicide resistance. Such model studies can contribute to the development of guidance on, for example, the maximum number of applications for a MOA (van den Bosch *et al*., 2015a). It should be noted that, for the purposes of this study, it has been assumed that the QoI type fungicide and its low‐risk mixing component are the only fungicides available, and that effective control and maximum effective life have to be achieved with these fungicides only. If more MOAs are available for a treatment programme, the optimal spray programme should use the available MOAs either in alternation or in mixtures. Using more MOAs than the high‐risk and the low‐risk fungicides modelled here will lead to a longer effective life of the high‐risk fungicide (van den Bosch *et al*., 2014a,b). Thus, the results presented here give an upper boundary in the use of a high‐risk fungicide (if more MOAs are used the high‐risk fungicide dose can generally be reduced), and the results suggest that less than or equal to two applications is the optimal application programme. However, in practice, wider considerations may modulate this guidance. If the limit on the maximum number of treatments for a MOA is set at a level below the number of treatments required by the grower for maximum gross economic margin, then there are two possible outcomes: either growers restrict the total number of fungicide treatments and have to accept a lower economic gain (and lower food production) in the short term, in the expectation of prolonged effective life repaying this in the long term; or the grower uses a different MOA for the additional sprays required meaning there must be sufficient effective other MOAs available. If this is not the case, the restriction pushes the growers away from the use of mixtures and towards the use of alternations. Although both mixtures and alternations are effective resistance management strategies, it is unclear whether alternations will provide a better balance between efficacy and selection than mixtures (Hobbelen *et al*., 2013; van den Bosch *et al*., 2015b).

## Supporting information


**Figure S1** Percentage healthy area duration (HAD) loss versus selection ratio in the first year.

**Data S1** Summary of fungicide dynamics for a fungicide mixture.

**Data S2** The pyraclostrobin and chlorothalonil dose–response curves.
