# Supplementary figures and images for: Dose and number of applications that maximize fungicide effective life exemplified by Zymoseptoria tritici on wheat – a model analysis
Source: Plant Pathol. 2016 Jun 10;65(8):1380–9. doi: 10.1111/ppa.12558 (PMC5027893; doi:10.1111/ppa.12558)

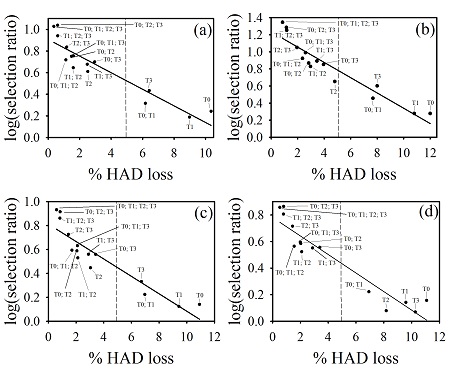

Supplement: Supplementary file 1 — Figure S1 Percentage healthy area duration (HAD) loss versus selection ratio in the first year. [file PPA-65-1380-s001.jpg]
