# Supplementary material for: Dose and number of applications that maximize fungicide effective life exemplified by Zymoseptoria tritici on wheat – a model analysis
Source: Plant Pathol. 2016 Jun 10;65(8):1380–9. doi: 10.1111/ppa.12558 (PMC5027893; doi:10.1111/ppa.12558)
Supplement: Supplementary file 2 — Data S1 Summary of fungicide dynamics for a fungicide mixture. [file PPA-65-1380-s002.docx]

**Supporting information S1**

**Summary of fungicide dynamics for a fungicide mixture**

The paper by van den Berg *et al*. (2013) considered only the effects of a single high-risk fungicide and therefore for this paper the model was extended to include the effects of a low-risk fungicide in mixture with the high-risk fungicide.

**Fungicide dynamics**

Fungicide treatments affect the pathogen life-cycle parameter values whereby the high-risk fungicide affects the transmission rate (included in $\rho_{S}$) and the length of the latent period $\left( 1/{m\delta_{S}} \right)$ and the low-risk fungicide affects the transmission rate of both strains (included in $\rho_{S}$ and $\rho_{R}$). The high- and low-risk fungicides are assumed to have independent actions, so that the total effect of the mixture is given by multiplying the fractions of the population surviving the individual products (Hobbelen *et al*., 2011b). The latent period and the compound parameter are then given by:

$\rho_{S}\left( t \right)=\rho\left[ 1-\alpha_{\rho,max}\left( 1-e^{-{\kappa_{\rho_{h}}d}_{h}(t)} \right) \right]\left[ 1-\beta_{\rho,max}\left( 1-e^{-\kappa_{\rho_{l}}d_{l}(t)} \right) \right]$

$\rho_{R}\left( t \right)=\rho\left[ 1-\beta_{\rho,max}\left( 1-e^{-\kappa_{\rho_{l}}d_{l}(t)} \right) \right]$ (1)

$\delta_{S}\left( t \right)=\delta\left[ 1-\alpha_{\delta,max}\left( 1-e^{-{\kappa_{\delta_{h}}d}_{h}(t)} \right) \right]$

$\delta_{R}\left( t \right)=\delta$

with $\alpha_{\rho,max}$ and $\alpha_{\delta,max}$ ($\beta_{\rho,max}$ and $\beta_{\delta,max}$) the maximum reduction in the target parameter and $\kappa_{\rho_{h}}$ and $\kappa_{\delta_{h}}$ ($\kappa_{\rho_{l}}$ and $\kappa_{\delta_{l}}$) the shape parameters of the dose-response curves for the high-risk (low-risk) fungicide and $d_{h}(t)$ and $d_{l}(t)$ the effective daily fungicide dose of the high-risk and low-risk fungicide, respectively.

The high-risk and low-risk fungicide dose, $D_{h_{n}}$ and $D_{l_{n}}$, respectively, that arrives at leaf *n* depends on total dose sprayed, $D_{0}$ and the probability of not being intercepted by any of the leaves above leaf *n*, such that

$D_{p_{n}}=D_{p_{0}}\left( 1-e^{-\tau A_{n}(t_{spray})} \right)e^{-\tau\sum_{j=1}^{n-1} A_{j}(t_{spray})}, \text{with} p=\{h,l\}$ (2)

whereby $\tau$ is a measure for the average projected area of the leaves and stem onto a horizontal surface. The applied active substance of the fungicide will decay over time, resulting in an effective daily dose concentration $d(t)$ on leaf *n* of

$d_{p}\left( t \right)=\frac{D_{p_{n}}}{A_{n}}e^{-\nu_{p}(t-t_{spray})}, \text{with} p=\{h,l\}$ (3)

where $\nu_{p}$ is the decay rate of the active substance in fungicide *p*.

The effect of the spray event on the actual density of the infectious lesions on the rosette leaves is delayed by a latent period and can be modelled by

$X\left( t \right)=\left\{ \begin{aligned} X_{0}\left( 1-\beta_{X,max}\left( 1-e^{-\kappa_{X_{l}}d_{l}\left( t-\frac{1}{\delta} \right)} \right) \right) \\ \left( \left( 1-\eta\right)\left( 1-\alpha_{X,max}\left( 1-e^{-\kappa_{X_{h}} d_{h}(t-1/\delta)} \right) \right)+\eta\right), t<T_{decline} \\ X_{0}e^{-\psi\left( t-T_{decline} \right)}\left( 1-\beta_{X,max}\left( 1-e^{-\kappa_{X_{l}}d_{l}\left( t-\frac{1}{\delta} \right)} \right) \right) \\ \left( \left( 1-\eta\right)\left( 1-\alpha_{X,max}\left( 1-e^{-\kappa_{X_{h}} d_{h}(t-1/\delta)} \right) \right)+\eta\right), T_{decline}\leq t<T_{inoc death} \end{aligned} \right.$(4)

where $\eta$ is the fraction of infectious lesions on the rosette leaves that is resistant, $\alpha_{X,max}$ ($\beta_{X,max}$) is the maximum density reduction of the inoculum on the rosette leaves due to a spray event with a high-risk (low-risk) fungicide and $\kappa_{X_{h}}$ ($\kappa_{X_{l}}$) is the dose-response parameter for the lesion density on the rosette leaves and the high-risk (low-risk) fungicide.

Spraying the lower leaves results in a temporary decline of the susceptible lesion population on these leaves, which causes an increase in the fraction of resistance, $\eta$, in the lesion density on the rosette leaves which becomes evident a latent period after the spray application, i.e. at time $t_{s}+\delta$. The new fraction can be calculated from

$\eta\left( t_{S}^{+}+1/\delta\right)= \frac{\eta\left( t_{S}^{-}+1/\delta\right)X\left( t_{S}^{-}+1/\delta\right)}{\left( 1-\eta\left( t_{S}^{-}+1/\delta\right) \right)X\left( t_{S}^{-}+1/\delta\right)\left( 1-\alpha_{X,max}\left( 1-e^{-\kappa_{X_{h}}d_{h}(t_{s}} \right) \right)+\eta\left( t_{S}^{-}+1/\delta\right)X\left( t_{S}^{-}+1/\delta\right)}$ (5)

where superscripts ‘−‘ and ‘+’ refer to an infinitesimal small time step before or after the time of the spray application, respectively.
