# Supplementary material for: Dose and number of applications that maximize fungicide effective life exemplified by Zymoseptoria tritici on wheat – a model analysis
Source: Plant Pathol. 2016 Jun 10;65(8):1380–9. doi: 10.1111/ppa.12558 (PMC5027893; doi:10.1111/ppa.12558)
Supplement: Supplementary file 3 — Data S2 The pyraclostrobin and chlorothalonil dose–response curves. [file PPA-65-1380-s003.docx]

**Supporting information S2**

**The pyraclostrobin and chlorothalonil dose–response curves**

The dose–response curves for the QoI fungicide (high-risk) and chlorothalonil (low-risk) fungicide are presented in Figure 1.

For chlorothalonil and pyraclostrobin data were obtained from Lockley & Clark (2005). Disease severities were assessed on leaves 3 and 4 (or 1 and 2) 3 weeks (or 6 weeks) after a single spray at growth stage (GS) 32 (Zadoks *et al.*, 1974). The average of these observed disease severities was used as an estimate of the disease severity on the upper leaves. The data points were then rescaled for a common disease severity in the absence of fungicides of 28%. Detailed information about fitting the model to the dose–response curve to estimate the dose–response curve parameters can be found in van den Berg *et al*. (2013) and the resultant model parameters are given in Table 2.
